# Preparation, characterization, and evaluation of chitosan-thymol nanoparticles for antibacterial and antibiofilm activities against catheter-associated uropathogenic *Escherichia coli*

**DOI:** 10.1186/s12896-026-01147-9

**Published:** 2026-04-01

**Authors:** Yasaman Dini, Mohamad Javad Mirzaei-Parsa, Mehrdad Halaji, Farzin Sadeghi, Abazar Pournajaf, Hoda Shirafkan, Mehdi Rajabnia

**Affiliations:** 1 Student Research Committee, Babol University of Medical Sciences, Babol, Iran; 2 Pathology and Stem Cell Research Center, Kerman University of Medical Sciences, Kerman, Iran; 3 Infectious Diseases and Tropical Medicine Research Center, Health Research Institute, Babol University of Medical Sciences, Babol, Iran; 4 Cellular and Molecular Biology Research Center, Health Research Institute, Babol University of Medical Sciences, Babol, Iran; 5 Social Determinants of Health Research Center, Health Research Institute, Babol University of Medical Sciences, Babol, Iran

**Keywords:** UPEC, UTIs, Biofilm, Thymol, Real-time PCR, MTT

## Abstract

Uropathogenic *Escherichia coli* (UPEC) is a major cause of urinary tract infections (UTIs) and is capable of forming biofilms on inert surfaces, contributing to persistent and recurrent infections. Given the limitations of conventional antibiotics, alternative strategies are needed to control multidrug-resistant (MDR) UPEC. This study aimed to evaluate the antibacterial and antibiofilm effects of thymol-loaded chitosan nanoparticles (TLCNPs) against MDR UPEC isolates urinary catheters. TLCNPs were prepared using the ionotropic gelation method and characterized by scanning electron microscopy (SEM), dynamic light scattering (DLS), and Fourier-transform infrared spectroscopy (FTIR). The minimum inhibitory concentration (MIC) and minimum bactericidal concentration (MBC) were determined using the broth microdilution method. Antibiofilm activity was assessed by the microtiter plate crystal violet assay. The relative expression of fimA and fimH genes was evaluated using real-time PCR. Cytotoxicity was assessed by the MTT assay on a human bladder carcinoma cell line (EJ138). The results showed that TLCNPs exhibited antibacterial activity against MDR UPEC isolates, with MIC and MBC values of 625 µg/mL. Biofilm formation was reduced by up to 76.2 ± 0.6% at ½ MIC concentration. Although a decrease in the relative expression of fimA and fimH genes was observed following treatment with TLCNPs, these changes were not statistically significant. Cytotoxicity assays indicated that more than 70% of EJ138 cells remained viable after 24 h, and over 50% after 48 h at ½ MIC. Overall, this study evaluated the antibacterial, antibiofilm, and preliminary safety profile of thymol-loaded chitosan nanoparticles against MDR UPEC, providing experimental data for their further investigation as a potential adjunct approach for biofilm-associated UTIs.

## Clinical trial number

Not applicable.

## Introduction

Urinary tract infections (UTIs) represent one of the most significant public health challenges globally. Uropathogenic *E. coli* (UPEC) is part of the Extra-intestinal Pathogenic *E. coli* (ExPEC) group, which predominantly originates from the intestinal microbiota and accounts for 65–90% of UTI cases [1]. Catheter-associated urinary tract infections (CAUTIs) are a significant cause of nosocomial infections, with indwelling catheters responsible for about 80% of these infections [2]. CAUTIs are able to cause serious problems, such as pyelonephritis, Gram-negative bacteremia, endocarditis, vertebral osteomyelitis, septic arthritis, endophthalmitis, and meningitis which are linked to enhancement of mortality, extended hospital stays and raised medical expenses [3]. UPEC has the ability to form biofilms on the surface of urinary catheters these biofilms consist of bacterial clusters that are attached to each other and to the catheter surface by structures like pili and fimbriae, and are encased in a self-produced extracellular matrix. The formation of biofilm not only contributes to the persistence of the bacteria on surfaces but also enhances their resistance to both the immune response and treatments such as antibiotics [4]. Attachment is the initial step in biofilm formation, where fimbriae, extracellular protein structures, play a crucial role. UPECs utilize several types of fimbriae, including type 1, P, S, and F1C fimbriae, to facilitate the attachment to various surfaces. These fimbriae enable UPECs to adhere to bladder cells, which is essential for the establishment of urinary tract infections [5]. Biofilm formation is a complex process involving various genes and regulatory mechanisms. Type 1 fimbriae (T1F), the most common fimbriae in UPECs, are encoded by the fim operon, which consists of nine genes, from *fimA* to *fimI*. While FimA serves as the main structural element, proteins such as FimG and FimF are added, with FimH at the tip. The expression of these fimbriae is crucial for UPEC’s ability to attach to surfaces, although it is not always expressed. The roles of genes like *fimC* and *fimD* are essential in transporting and assembling these fimbriae, ultimately contributing to the formation of biofilms [5]. FimH is a crucial virulence factor in UPEC that facilitates attachment to the host cells, particularly urothelial cells, through its interaction with D-mannose-containing receptors such as uroplakins. This interaction is essential for colonizing and establishing intracellular bacterial communities (IBCs) in the bladder. Additionally, FimH contributes to biofilm formation and the development of antibiotic resistance, playing a remarkable role in the persistence of UPEC infections in the urinary tract [5–7]. Antibiotic resistance and infectious diseases represent major challenges in healthcare, with the World Health Organization recognizing them as a global crisis [8]. The widespread use of antibiotics in treating urinary tract infections, even for short durations, significantly contributes to the rapid development of antibiotic resistance in uropathogenic strains, particularly UPEC. Recent studies indicate that pathogenic *E. coli* strains are progressively showing resistance to a wide range of antibiotics, like β-lactams, tetracyclines, fluoroquinolones, and aminoglycosides [9]. In response to this growing problem, recent advancements in antimicrobial nanomaterials are being explored as a potential solution. Herbal compounds (such as extracts, essential oils, gums, seeds, and fruits) have significant antimicrobial properties and minimal toxicity, which can provide a very suitable strategy to combat infectious diseases caused by drug-resistant bacteria [3].

Thymol is a natural bioactive agent widely used in traditional medicine and the food industry because of its medicinal features, which includes antimicrobial, anti-inflammatory, and anticancer effects. However, its usage is limited by challenges such as poor bioavailability, relatively low solubility. One of the important methods that can be used to overcome the limitations of herbal compounds is encapsulation, which involves coating bioactive materials and compounds at the nanoscale [10].

Natural polymers, compared to bio-resourceable synthetic polymers, are ideal materials for sustained release formulations due to their exceptional and safe properties, including biocompatibility, biodegradability, abundance in nature, and cost-effectiveness. Among natural polymers, chitosan has been widely used in the design of nanocarriers [11]. The integration of nanotechnology into drug formulations can improve the characteristics of conventional drugs, enhancing their absorption, specificity for target sites, and facilitating their attachment to drug delivery systems [7]. Ttherefore, considering the increasing prevalence of antibiotic-resistant UPEC strains and the need for alternative antimicrobial approaches, the present study aimed to evaluate the antibacterial activity of thymol-loaded chitosan nanoparticles (TLCNPs) against multidrug-resistant UPEC isolates obtained from urinary catheters. The study focused on assessing the antibacterial and antibiofilm potential of TLCNPs, examining their effects on the relative expression of selected biofilm-associated genes (fimA and fimH), and preliminarily evaluating their cytotoxicity using a human bladder carcinoma cell line to determine the in vitro safety profile. We hypothesized that treatment with sub-MIC concentrations of TLCNPs would inhibit bacterial growth and biofilm formation, reduce fimA and fimH expression, and maintain cell viability above 70% after 24 h of exposure.

## Materials and methods

### Preparation of thymol- loaded chitosan nanoparticles (TLCNPs)

The chitosan nanoparticles were prepared based on the ionotropic gelation method [12]. Briefly, 0.2% (w/v) low-molecular-weight chitosan (Sigma-Aldrich, Germany; product code 448869-50G), characterized by a molecular weight of 50,000–190,000 Da (determined by viscometry) and a viscosity of 20–300 cP (measured as a 1% w/v solution in 1% acetic acid at 25 °C), was dissolved in 0.1% (v/v) acetic acid under gentle stirring at ambient temperature until a clear, homogeneous solution was achieved. Thymol powder (Sigma-Aldrich, Germany) at a concentration of 2 mg/ml was dissolved in absolute ethanol, after that 1% (v/v) Tween 80 was added to the thymol solution. TLCNPs nanoparticles were obtained by adding the thymol solution to the chitosan solution (pH adjusted to 5.5) under magnetic stirring at 1000 rpm. Subsequently, 1 ml of an aqueous solution of tripolyphosphate (TPP, 0.2% w/v) as the cross-linker was added dropwise to 3 ml of chitosan-thymol solution and continuously stirred at room temperature for 1 h. To reduce the particle size, the prepared suspensions were probe-sonicated (at 80% amplitude) up to 10 min with a 1 min pause every 2 min of sonication.

### Encapsulation efficiency

To separate free thymol and thymol encapsulated in chitosan, the prepared solution was centrifuged at 14,000 rpm for 10 min at 4 °C. The supernatant, containing free thymol, was separated from the precipitate containing thymol-loaded chitosan nanoparticles. The absorbance of the free drug was then measured using a UV spectrophotometer at a wavelength of 276 nm, which is specific to thymol. Finally, the obtained value was used in following equation [13]:

$$\eqalign{ & Encapsulation{\kern 1pt} Efficiency\,(\% ) \cr & = \left( {{\matrix{ Total\>thymol\>loading\>concentration \hfill \cr - \>Free\>thymol\>concentration \hfill \cr} \over {Total\>thymol\>loading\>concentration}}} \right) \times 100 \cr}$$

### Characterization of TLCNPs

The morphology and size of the TLCNPs were analyzed employing a scanning electron microscope (TESCAN, Czech Republic). Dynamic light scattering (DLS, Cordouan technologies, France) was used to ascertain hydrodynamic size of particles [14]. Fourier transform infrared spectroscopy (FTIR, AVATAR, Thermo, America) was employed to identify the functional groups of TLCNPs within a wavelength range of 400–4000 cm⁻¹.

### Bacterial isolates

*E. coli* ATCC^®^ 25,922™ and four MDR isolates from phylogenetic group B_2_ among the UPEC isolates (urinary catheters) confirmed in a previous study, were obtained [15].

### Detection of *fimA* and *fimH* genes

Bacterial DNA was extracted from fresh colonies, as described previously [16]. The polymerase chain reaction (PCR) was carried out to detect the presence of *fimA* and *fimH* genes using the specific primers in Table 1. The conditions for PCR amplification were: initial denaturation at 94 °C for 5 min, followed by 30 cycles of denaturation at 94 °C for 30 s, primer annealing at Table 1 for 30 s, extension at 72 °C for 30 s, and the final extension 72 °C for 5 min.

**Table 1:** Primer sequence of target genes

Target Genes	Sequence Primer (5′➔3′)	Amplification Size(bp)	Annealing Temperature (°C)	References
*fimA*-F	ACTCTGGCAATCGTTGTTCTGTCG	144	56	[17]
*fimA*-R	ATCAACAGAGCCTGCATCAACTGC			
*fimH*-F	GCTGTGATGTTTCTGCTCGT	168	51	
*fimH*-R	AAAACGAGGCGGTATTGGTG			
*gapA*-F	ACTTACGAGCAGATCAAAGC	170	49	[18]
*gapA*-R	AGTTTCACGAAGTTGTCGTT			

### GenBank accession numbers

Moreover, sequences of the *fimA* and *fimH* genes were deposited in the NCBI database with the accession numbers PP933805 and PP933806.

### Investigating antibacterial properties of TLCNPs

#### Measurement of minimum inhibitory concentration (MIC) and minimum bactericidal concentration (MBC)

The MIC and MBC of both thymol and TLCNPs were determined using the broth microdilution method in a 96-well microplate Based on the CLSI standard [19]. For both agents, 100 µl of Muller Hinton Broth (MHB) was added to each well of the microplate. The solutions were serially diluted in the range of 10–0.01 mg/ml for thymol and 2–0.002 mg/ml for TLCNPs. Each well was inoculated with 10 µl of a bacterial suspension consisting of 1.5 × 10^6^ CFU/ml. Negative controls were wells without bacteria, and positive controls were wells without antimicrobial agents. The microplates were incubated at 37 °C for 24 h. The MIC was determined as the lowest concentration of the antimicrobial agent that inhibited visible growth of the bacteria.

For the MBC assay, after incubation, 10 µl from each well was cultured onto Muller Hinton Agar (MHA), and the plates were incubated at 37 °C for 24 h. The MBC was defined as the lowest concentration at which no bacterial growth was observed [20].

#### Antibiofilm potential of TLCNPs

The antibiofilm activity of TLCNPs was assessed using the crystal violet staining assay [21]. Briefly, 100 µL of bacterial culture in Tryptic Soy Broth (TSB) containing 0.2% at a concentration of 1.5 × 10^6^ CFU/mL was inoculated into the wells of a 96-well plate. Subsequently, 100 µL of sub-MIC concentrations of TLCNPs ($$\:\raisebox{1ex}{$1$}\!\left/\:\!\raisebox{-1ex}{$2$}\right.$$ MIC, $$\:\raisebox{1ex}{$1$}\!\left/\:\!\raisebox{-1ex}{$4$}\right.$$ MIC, $$\:\raisebox{1ex}{$1$}\!\left/\:\!\raisebox{-1ex}{$8$}\right.$$ MIC, and $$\:\raisebox{1ex}{$1$}\!\left/\:\!\raisebox{-1ex}{$16$}\right.$$ MIC) was added to the wells. Positive control wells contained TSB with bacterial suspension, and negative control wells contained only TSB without bacteria. The plates were incubated at 37 °C overnight. Following incubation, the cultures were aspirated, and the wells were washed twice with sterile distilled water to remove non-adherent cells. The plates were air-dried, and the adherent cells were fixed with absolute methanol for 15 min. Adherent cells were then stained with 0.2% crystal violet for 15 min. Excess dye was removed, and the wells were washed thrice with 100 µL of sterile distilled water. Finally, the optical density (OD) of the stained biofilm was measured at 570 nm using a micro-ELISA auto reader after solubilizing the dye with 125 µL of 30% acetic acid. Wells with TSB alone served as blanks.

The percentage of biofilm inhibition was calculated using the following equation:

$$\begin{aligned}&Biofilm\,Inhibition\,(\%)\cr&\quad=100\,-\,(\:\frac{\:treated\:well\:absorbance}{\:\:untreated\:well\:\:absorbance}\times\:100)\end{aligned}$$

#### RNA extraction and cDNA synthesis

A bacterial suspension of 10⁹ CFU/mL was prepared for each isolate in TSB containing 0.2% glucose and ½ MIC concentration of TLCNPs. For the control, the same medium without NPs was used. Samples were incubated overnight at 37 °C, and after incubation, the supernatants were discarded. Total RNA was extracted using the Total RNA Extraction Kit (Viragen Co., Iran) following the manufacturer’s instructions.

The RNA quantity was assessed using a Nanodrop spectrophotometer by measuring the absorbance at 260 nm, while the purity was determined by the 260/280 nm absorbance ratio. An RNA purity ratio close to ~ 2.0 was considered acceptable. The quality of the extracted RNA was further verified by electrophoresis on a 1% agarose gel. Genomic DNA contamination was removed using RNase-free DNase I (Viragen Co., Iran), according to the manufacturer’s guidelines. After DNase treatment, the RNA samples were evaluated by electrophoresis on a 1% agarose gel to confirm DNA removal. Complementary DNA (cDNA) was synthesized using the cDNA Synthesis Kit (Parstous Co., Iran) as per the manufacturer’s instructions.

#### Effect of TLCNPs on gene expression of *fimA* and *fimH*

The expression levels of *fimA* and *fimH* genes were determined using quantitative real-time PCR (qPCR) with the ABI StepOnePlus Real-Time PCR System (Applied Biosystems, USA). The reactions were carried out using SYBR Green Master Mix (Ampliqon, Denmark) in a total volume of 20 µL, which included 10 µL of SYBR Green Master Mix, one µL of forward primer, one µL of reverse primer, two µL of cDNA, and six µL of nuclease-free water.

The qPCR thermal cycling conditions consisted of an initial denaturation step at 95 °C for 15 min, followed by 40 cycles of denaturation at 95 °C for 15 s, annealing at the optimized temperature for 30 s, and extension at 72 °C for 30 s. The housekeeping gene *gapA* was employed as the internal control. Primer sequences are listed in Table 1.

The relative expression levels of the target genes were analyzed using the comparative cycle threshold (Ct) method 2 ^(−ΔΔCt)^. All experiments were performed in triplicate for statistical reliability.

### Cytotoxicity of TLCNPs

The cytotoxicity of TLCNPs against human bladder carcinoma was evaluated using the MTT [3-(4,5-dimethylthiazol-2-yl)-2,5-diphenyltetrazolium bromide] assay. The EJ138 cell line was purchased from the cell bank of Pasteur Institute of Iran. A total of 7 × 10^5^ EJ138 cells were seeded into 96 well plates and incubated for 24 h. Then, EJ138 cells were treated with varying concentrations of TLCNPs ($$\:\raisebox{1ex}{$1$}\!\left/\:\!\raisebox{-1ex}{$2$}\right.$$ MIC, $$\:\raisebox{1ex}{$1$}\!\left/\:\!\raisebox{-1ex}{$4$}\right.$$ MIC, $$\:\raisebox{1ex}{$1$}\!\left/\:\!\raisebox{-1ex}{$8$}\right.$$ MIC, and $$\:\raisebox{1ex}{$1$}\!\left/\:\!\raisebox{-1ex}{$16$}\right.$$ MIC). After treatment, the 96 well plates were incubated for 24 h and 48 h then, 50 µl of MTT dye was added into wells and incubate for 4 h. Finally, purple color formazone crystals were dissolved in dimethyl sulfoxide (DMSO) and the OD was read at 570 nm using ELISA reader. The cell toxicity was determined using the following equation [22]:

$$\begin{aligned}&Cytotoxicity\,assay\cr&\quad=[(\:\frac{\:treated\:cells\:\:absorbance\:-\:blank\:absorbance}{control\:\:absorbance\:\:-\:blank\:absorbance})\times100]\end{aligned}$$

### Statistical analysis

Statistical analysis was conducted using GraphPad Prism software version 9.5.1. The real-time PCR results were analyzed using a t-test, while the MTT results and biofilm inhibition effects were analyzed using a one-way ANOVA. Statistical significance was defined as a P-value of less than 0.05. All experiments were performed in triplicate, with a minimum of three repeats.

## Results

### Characterization of TLCNPs

**Fig. 1:**
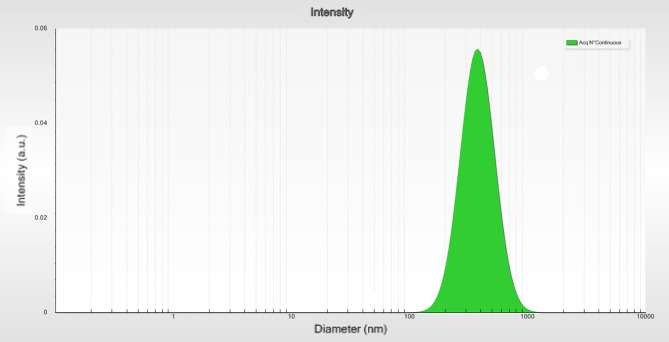
DLS analysis reveals an average hydrodynamic diameter of 317.39 nm

DLS analysis indicated that the TLCNPs had an average hydrodynamic diameter of approximately 317.39 nm (Fig. 1).

**Fig. 2:**
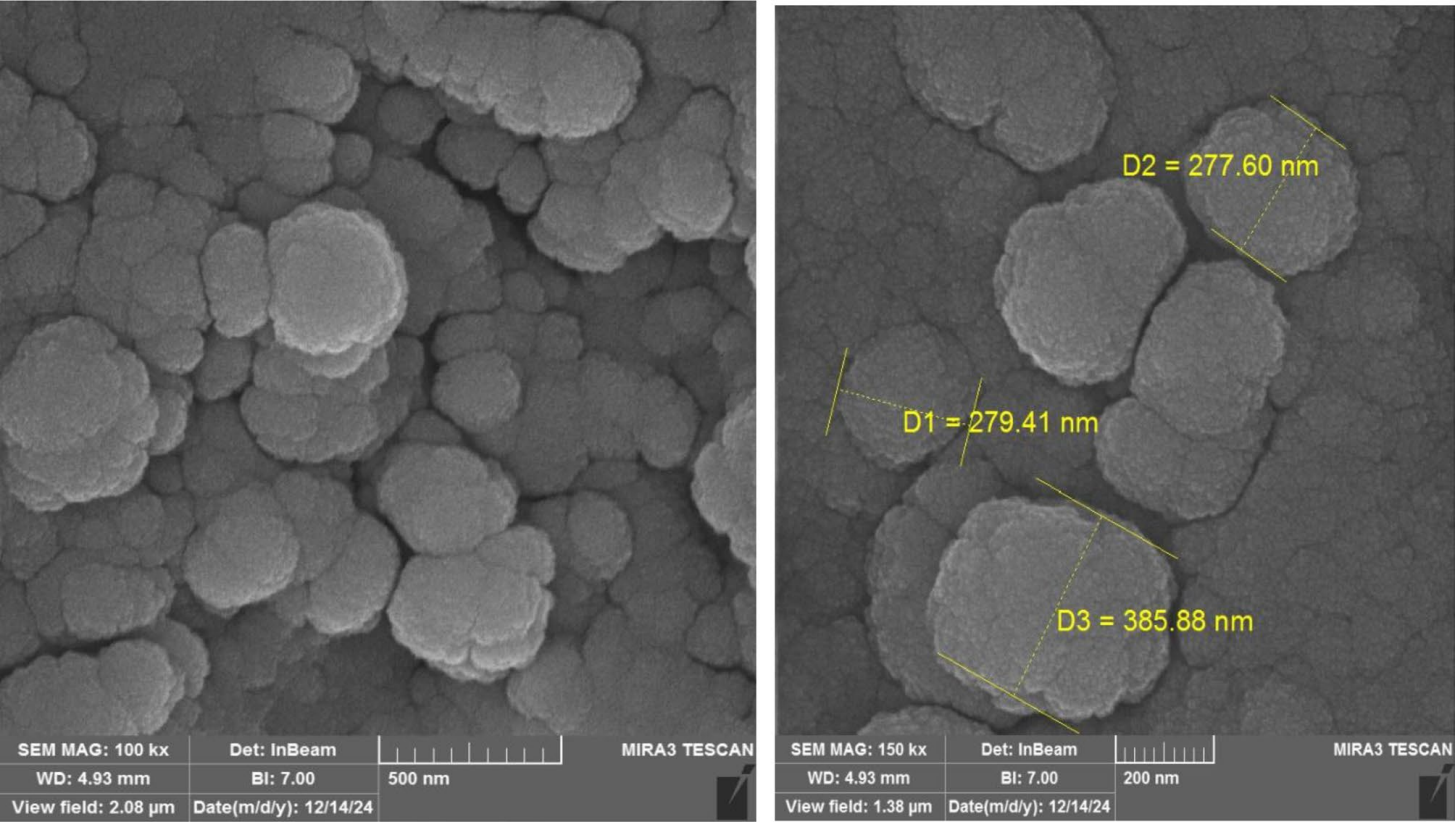
Morphological and size characterization of TLCNPs. SEM images depict TLCNPs as generally spherical and uniform, with an average size of 307 ± 34 nm

The morphology and size of the TLCNPs were assessed using SEM, indicating a generally spherical and uniform shape with an average diameter of 307 ± 34 nm, as shown in Fig. 2. Encapsulation efficiency (EE) is one of the parameters that directly reflects the concentration of the active ingredient, thymol, entrapped in the chitosan matrix. The encapsulation efficiency of TLCNP was obtained 70 ± 0.53%.

**Fig. 3:**
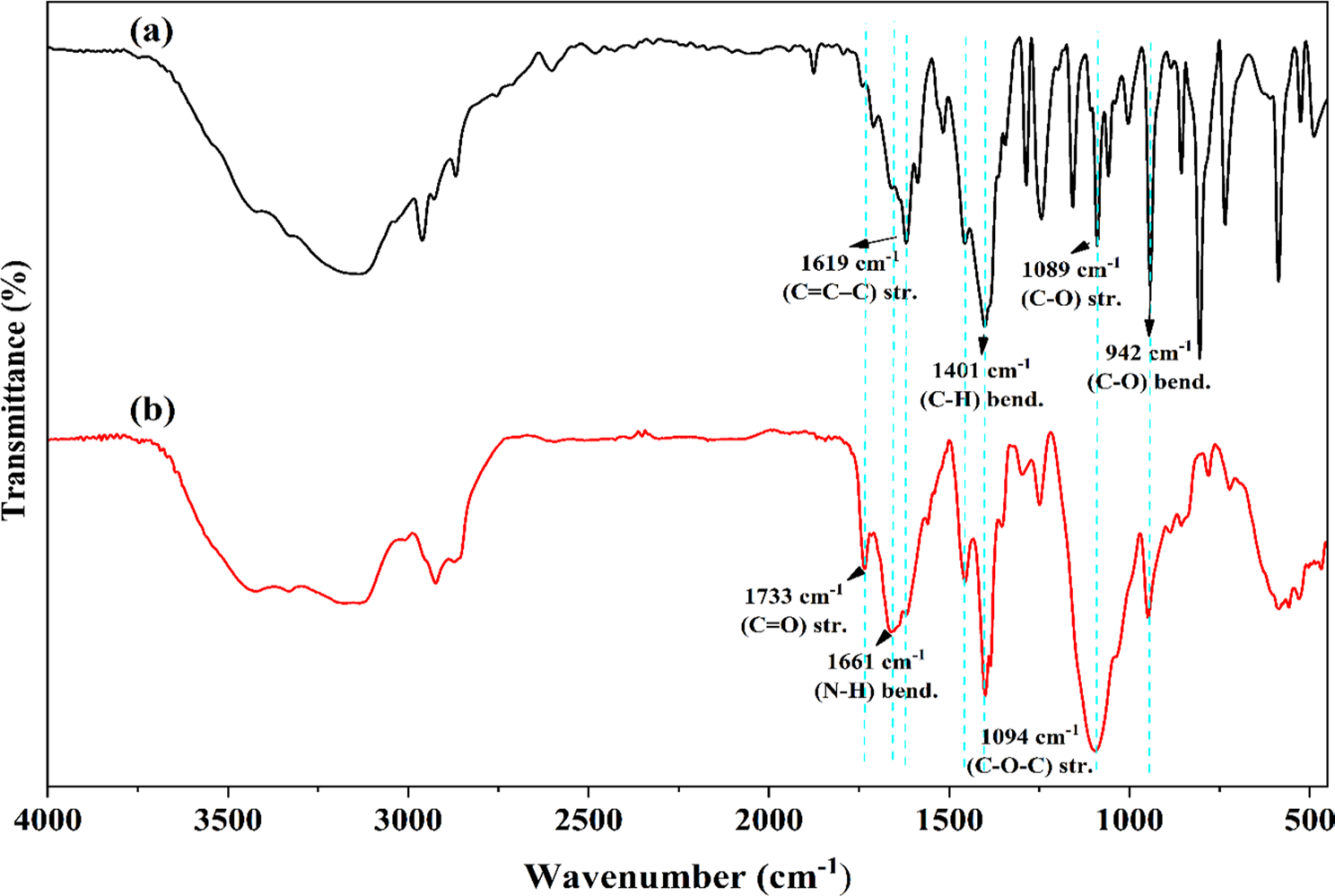
FT-IR spectrum of (**a**) thymol and (**b**) TLCNPs

The FT-IR spectra of thymol and TLCNPs are presented in Fig. 3. The FT-IR spectrum of pure thymol shows characteristic bands such as O–H stretching at 3330 cm⁻¹, aromatic C–H at 3137 cm⁻¹, aliphatic C–H at 2961 and 2868 cm⁻¹, and C = C–C stretching at 1619 and 1587 cm⁻¹. In the FT-IR spectrum of TLCNPs, these peaks appear at slightly different positions—O–H at 3390 cm⁻¹, aromatic C–H at 3174 cm⁻¹, and C = C–C at 1618 cm⁻¹—indicating a shift due to interactions within the nanoparticle matrix. The peaks at 1734 cm-¹ and 1661 cm-¹ corresponding to amide I (C = O) and N–H bending, respectively, showed the presence of chitosan in TLCNPs [23–25]. The observed shifts in thymol’s peaks, along with the presence of both thymol and chitosan signals in the encapsulated sample, confirm that thymol has been successfully incorporated into the chitosan nanoparticles and is interacting with the matrix structure, likely through hydrogen bonding and electrostatic interactions.

### Detection of *fimA* and *fimH* genes

PCR analysis demonstrated that all the samples contained *fimA* and *fimH* (genes, confirming their uniform presence across the studied isolates.

### Measurement of minimum inhibitory concentration (MIC) and minimum bactericidal concentration (MBC)

The MIC and MBC values of thymol and TLCNPs were determined to be 625 µg/mL for both MDR strains and ATCC^®^ 25,922™.

### Antibiofilm potential of TLCNPs

**Fig. 4:**
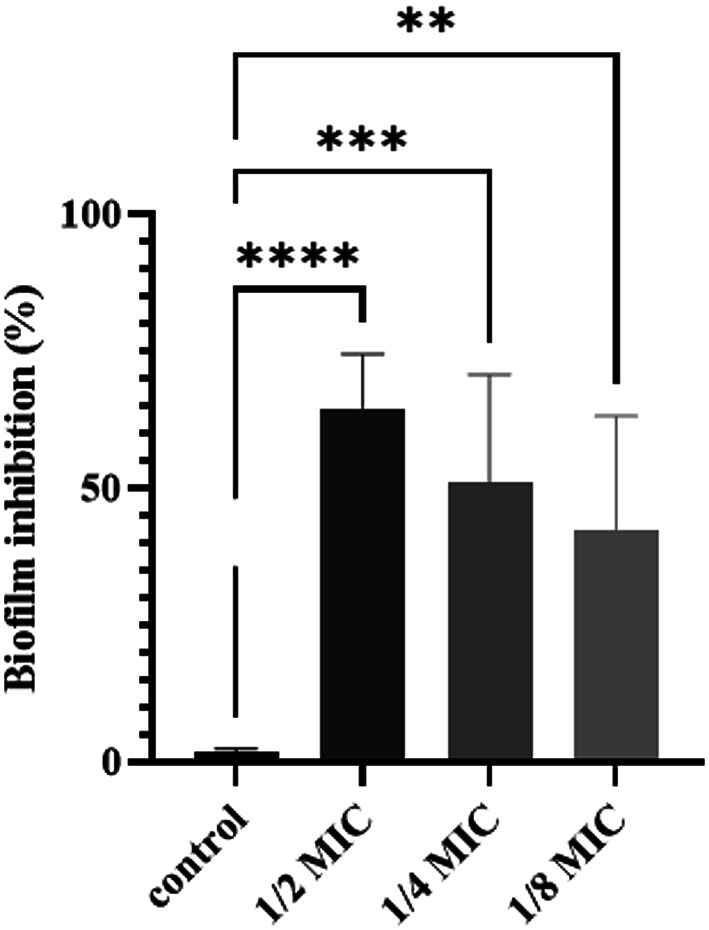
TLCNPs biofilm inhibition effect against isolates (** *p* < 0.01, ***p ˂ 0.001, **** p˂ 0.0001)

The ability of TLCNPs to inhibit biofilm formation at concentrations from 312 µg/mL to 78 µg/mL (MIC, ½ MIC, ¼ MIC, and ⅛ MIC) is presented in the Table 2. There was a significant correlation between the MIC and SUB-MIC concentrations of TLCNPs and biofilm inhibition, which is shown in the Fig. 4.

**Table 2:** Biofilm inhibition percentages by TLCNPs

Isolates	½ MIC 312 µg/ml Mean + SD	¼ MIC 156 µg/ml Mean + SD	⅛ MIC 78 µg/ml Mean + SD
Isolate 32	76.2 ± 0.6%	71.43 ± 0.7%	62 ± 0.07%
Isolate 33	50 ± 0.4%	37.5 ± 0.1%	25 ± 0.7%
Isolate 38	70.6 ± 0.6%	64.7 ± 0.1%	58.8 ± 0.7%
Isolate 47	61.5 ± 0.3%	23.7 ± 0.1%	15.4 ± 0.1%
ATCC25922	64.3 ± 0.2%	57.15 ± 0.2%	50 ± 0.6%

### Effect of TLCNPs on gene expression of *fimA* and *fimH*

**Fig. 5:**
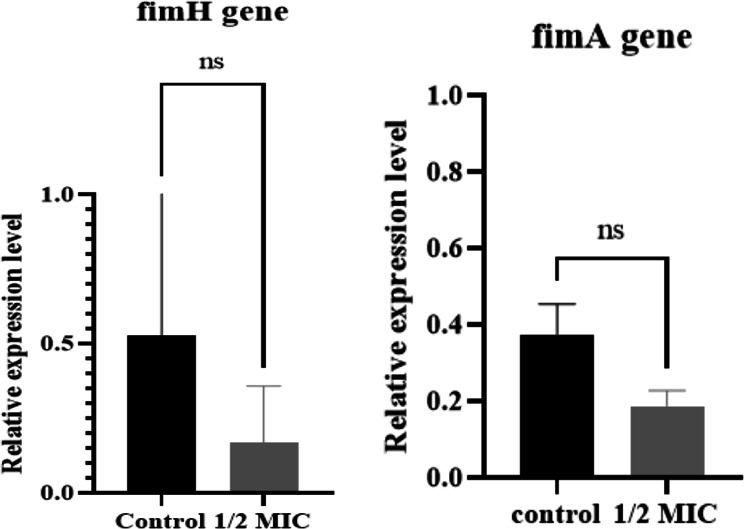
Effect of TLCNPs on the relative expression level of the *fimA* and *fimH* genes in all isolates (ns, not significant)

Relative expression of the *fimA* and *fimH* genes was tested in comparison to the reference gene *gapA*. Figure 5 shows the changes in *fimA* and *fimH* gene expression in all isolates exposed to a ½ MIC concentration of TLCNPs (312 µg/mL) compared to untreated controls. The expression ratios of *fimA* and *fimH* were not statistically significantly reduced after treatment, with p-values of 0.0520 and 0.2824, respectively. However, a non-significant downward trend was observed, with mean relative expression reduced by 81.25% and 83.13% for *fimA* and *fimH*, respectively. These findings suggest a potential effect of TLCNPs on *fimA* and *fimH* expression, which requires further investigation in larger studies.

### Cytotoxicity of TLCNPs

**Fig. 6:**
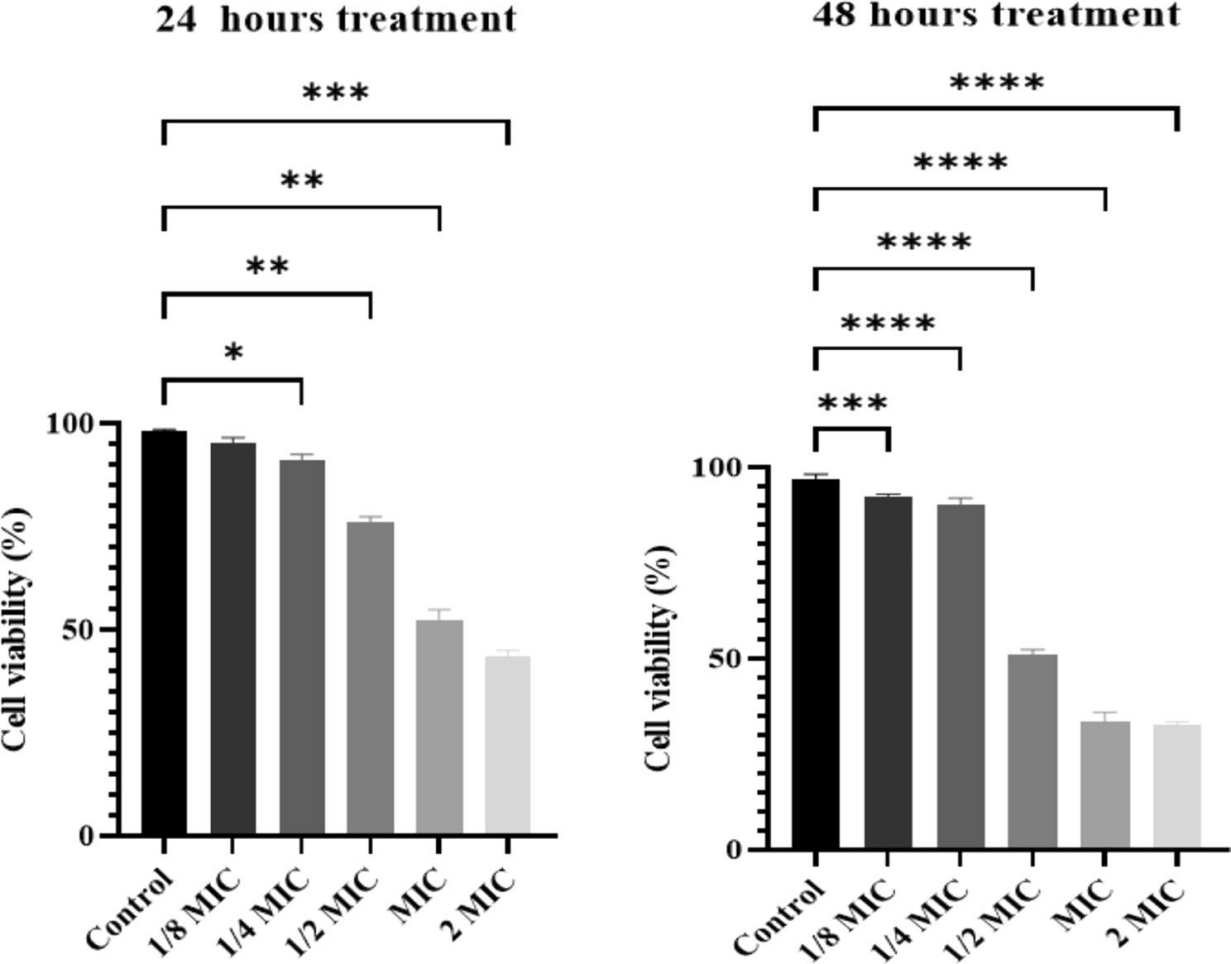
Cytotoxic effects of different concentrations of TLCNPs on EJ138 cells (* *p* < 0.05, ** *p* < 0.01, ***p ˂ 0.001, **** p˂ 0.0001)

The cytotoxicity of TLCNPs was evaluated using the MTT assay on EJ138 cells, and the percentage of cell viability was determined at various concentrations of TLCNPs (Fig. 6); as the concentration of TLCNPs augmented from 0 to 1250 µg/mL, cell viability diminished continuously at both time points. Viability progressively reduced with increasing concentration of TLCNPs, reaching approximately 33.5–45% at 1250 µg/mL (2MIC). Elevated concentrations of TLCNPs substantially decrease cell viability, demonstrating a dose-dependent cytotoxic impact on EJ138 cells (*p* < 0.05).

## Discussion

UPEC, the most common causative agent of UTIs, presents significant treatment challenges due to the emergence and spread of MDR bacterial strains. Biofilm formation, particularly in catheter-associated infections, exacerbates these challenges and often results in therapeutic failure. Nanoparticle-based antibacterial agents are emerging as promising tools for UTI treatment, offering enhanced stability, controlled drug release, and improved therapeutic efficacy compared to conventional therapies.

TLCNPs were prepared using the ionic gelation method. The interaction between positively charged chitosan and negatively charged TPP, a polyanionic cross-linking agent, led to the formation of thymol nanoparticles. During the formulation process, the amino groups of chitosan are protonated at low pH, and TPP, which electrostatically binds to these amine groups, creates chitosan nanoparticles via ionic bonding. The size of chitosan nanoparticles primarily depends on factors such as stirring speed, polymer concentration (chitosan), and the cross-linking agent (TPP) [26].

As demonstrated in this study, chitosan with a concentration of 0.2% and a TPP-to-chitosan ratio of 1:3 was used, resulting in an encapsulation efficiency of 70 ± 0.53%. DLS analysis confirmed the particle size to be 317.39 nm. In the study by Jaya Balang et al. (2022), the nanoparticle size was reported as 282.5 nm with an encapsulation efficiency of 74.73 ± 0.08%. These differences may be attributed to variations in chitosan concentration and the TPP to chitosan ratio (1:3 in the current study vs. 1:4 in Jaya Balang’s study) [27]. Increased chitosan concentration can lead to higher solution viscosity, resulting in larger nanoparticles, while a lower TPP to chitosan ratio might reduce encapsulation efficiency and increase particle size [26]. Additionally, slight differences in measurement methods and experimental conditions (such as temperature and pH) could affect the final particle size [28]. Overall, the outcomes of this study highlight the impact of synthesis conditions on the characterizations of chitosan nanoparticles and encapsulation efficiency [29].

Sarwar et al. showed that Low molecular weight Chitosan–TPP NPs (196 and 394 nm) exhibited better antibacterial effects compared to High molecular weight Chitosan–TPP NPs (598 and 872 nm). These findings indicate a negative correlation between antibacterial activity and both particle size and molecular weight. Therefore, CS–TPP NPs can be considered a hopeful antimicrobial agent [30].

Nanoparticles with Their small size and high surface area-to-volume ratio allow better penetration of bacterial cell walls and effective interactions with bacterial membranes [31]. *E. coli* has a thinner peptidoglycan layer than Gram-positive bacteria, making it generally more susceptible to certain antimicrobial compounds that target the cell wall [31].

In this study, we determined that both MIC and MBC of thymol and TLCNP are 625 µg/mL. At first glance, encapsulation does not appear to enhance the MIC or the antibacterial properties. However, it should be considered that the amount of thymol present in encapsulated complexes is lower than in its free form at the same concentrations. In the study by Abdollahi et al. (2024), thymol nanogel with a size of 85 nm and a concentration of 5000 µg/mL, and camphor-thymol nanogel with a size of 135 nm and a concentration of 5000 µg/mL, exhibited 100% inhibitory effect upon the standard *E. coli* strain [32].

Based on the current research, TLCNPs at 0.3125 mg/mL (½ MIC) inhibited biofilm formation by more than 50% in UPEC isolates from phylogenetic group B_2_, which were capable of forming strong or moderate biofilms [33]. The highest biofilm inhibition rate (76.2 ± 0.6%) was observed at ½ MIC, while the lowest inhibition rate (15.4 ± 0.1%) was noted at ⅛ MIC. Similarly, Cai et al. (2022) reported that encapsulating basil essential oil in chitosan nanoparticles, with a particle size ranging from 198.7 to 373.4 nm, exhibited strong antibacterial and antibiofilm activities against *E. coli*. However, the MIC and MBC values of these basil-loaded nanoparticles were reported as 5 mg/mL and 10 mg/mL, respectively [34]. In the study by Sharma et al. (2023), chitosan in solution form was evaluated using the disk diffusion method, which demonstrated relatively small inhibition zones. The limited antimicrobial activity of chitosan is attributed to the presence of amino groups in its structure, which act as relatively weak cationic centers and result in reduced cell membrane permeabilization. However, incorporation of thymol into chitosan films with particle sizes ranging from 197 to 389 nm markedly enhanced their antimicrobial efficacy. This improvement is primarily due to the phenolic hydroxyl groups of thymol, which play a central role in the antimicrobial activity of the formulated films [35].

Biofilm formation is regulated by complex mechanisms including quorum sensing and extracellular matrix production, which are distinct from planktonic growth inhibition measured by MIC. Several studies have shown that biofilm inhibition can occur at sub-MIC levels, likely by disrupting initial adhesion, quorum sensing, or extracellular polymeric substance production, even when planktonic growth is not fully suppressed [36, 37].

According to this study, TLCNPs at a concentration of ½ MIC (0.3125 mg/mL) reduced the expression of both the *fimA* and *fimH* genes. While there was no statistically significant correlation between concentration and *fimH* (*p* = 0.2824) or *fimA* (*p* = 0.052) reduction, a gradual decrease in the expression of these genes was observed. Similarly, Jamalan et al. (2019) noted a reduction in *fimH* expression in UPEC treated with zinc oxide nanoparticles (19.53 µg/mL), but this result was also statistically insignificant (*p* = 0.203) [38]. Encapsulation of thymol in chitosan enables its controlled release, resulting in prolonged antibacterial effects [7]. Potentially, Continuous exposure to thymol reduces bacterial adhesion and the formation of type 1 fimbriae, ultimately decreasing the expression of *fimA* and *fimH* genes. However, these findings should be interpreted with caution, as the observed changes were not statistically significant and the present study represents a preliminary investigation. Therefore, no definitive mechanistic conclusions can be drawn at this stage, and further studies employing larger sample sizes and complementary functional assays are required to validate these observations and clarify the underlying mechanisms.

The EJ138 human bladder cancer cell line, which closely resembles human urinary tract epithelial cells, was used to evaluate cytotoxicity. After 24 h, over 70% of cells survived at a concentration of ½ MIC (0.3125 mg/mL), and after 48 h, more than 50% remained viable. Thymol is a natural compound with antibacterial and anticancer properties [39]. According to Li et al. (2017), thymol induces apoptosis in bladder cancer cells through the intrinsic pathway involving caspase-3/9 activation, cytochrome c release, and downregulation of antiapoptotic Bcl-2 proteins [31]. On the other hand, Akhtar et al. (2012) demonstrated that zinc oxide nanoparticles were more toxic to cancer cell lines than to normal cell lines [40]. The EJ138 human bladder cancer cell line was employed as an initial in vitro model for cytotoxicity assessment. Although cancer-derived cell lines can offer preliminary insights into cellular responses, they do not fully recapitulate the physiological characteristics of normal urinary tract epithelial cells. Consequently, the findings of this assay should be interpreted as an initial safety screening rather than a definitive evaluation of cytocompatibility. Further investigations using normal urothelial cell lines are warranted to more accurately characterize the biocompatibility of TLCNPs.

## Conclusion

In this study, the antibacterial and antibiofilm effects of thymol-encapsulated chitosan nanoparticles (TLCNPs) against uropathogenic Escherichia coli were evaluated. The in vitro results showed that these nanoparticles were able to inhibit bacterial growth and biofilm formation to a measurable extent. However, it should be noted that these findings are limited to in vitro conditions and cannot be directly extrapolated to clinical applications.

Further research is needed to elucidate the molecular mechanisms underlying the antibacterial and antibiofilm effects of thymol and chitosan. Furthermore, in vivo studies using appropriate animal models, such as mouse urinary tract infection models, are necessary to assess the efficacy, safety, and potential immunological responses. The stability, persistence, and performance of the nanoformulations, especially in practical applications such as catheter coatings, should also be systematically evaluated.

Limitations of this study include the relatively small number of bacterial isolates tested, lack of in vivo validation, and lack of long-term stability assessment. Overall, these findings provide preliminary evidence supporting the potential of TLCNPs as a nanotechnology-based approach for controlling UPEC and other drug-resistant bacterial infections, while emphasizing the need for further comprehensive studies to confirm their practical application and safety.

## Data Availability

The datasets used and/or analyzed during the current study are available from the corresponding author on reasonable request.

## Abbreviations

UTIs: Urinary tract infections
UPEC: Uropathogenic E. coli
ExPEC: Extra-intestinal Pathogenic E. coli
CAUTIs: Catheter-associated urinary tract infections
T1F: Type 1 fimbriae
IBCs: Intracellular bacterial communities
TLCNPs: Thymol- Loaded Chitosan Nanoparticles
TPP: Tripolyphosphate
DLS: Dynamic light scattering
FTIR: Fourier transform infrared spectroscopy
PCR: Polymerase chain reaction
MIC: Minimum inhibitory concentration
MBC: Minimum bactericidal concentration
MHB: Muller Hinton Broth
MHA: Muller Hinton Agar
TSB: Tryptic Soy Broth
OD: Optical density
qPCR: Quantitative real-time PCR
Ct: Cycle threshold
DMSO: Dissolved in dimethyl sulfoxide
EE: Encapsulation efficiency
